# Whole Exome Sequencing Confirms Molecular Diagnostics of Three Pakhtun Families With Autosomal Recessive Epidermolysis Bullosa

**DOI:** 10.3389/fped.2021.727288

**Published:** 2021-08-03

**Authors:** Fozia Fozia, Rubina Nazli, Nousheen Bibi, Sher Alam Khan, Noor Muhammad, Nafila Shakeeb, Saadullah Khan, Musharraf Jelani, Naveed Wasif

**Affiliations:** ^1^Department of Biochemistry, Institute of Basic Medical Sciences, Khyber Medical University, Peshawar, Pakistan; ^2^Department of Biotechnology and Genetic Engineering, Kohat University of Science and Technology, Kohat, Pakistan; ^3^Department of Bioinformatics, Shaheed Benazir Bhutto Women University, Peshawar, Pakistan; ^4^Dermatology Department, Services Hospital, Peshawar, Pakistan; ^5^Centre for Omic Sciences, Islamia College Peshawar, Peshawar, Pakistan; ^6^Institute of Human Genetics, University of Ulm, University of Ulm Medical Center, Ulm, Germany; ^7^Institute of Human Genetics, University Hospital Schleswig-Holstein, Kiel, Germany

**Keywords:** epidermolysis bullosa, exome sequence, COL17A1 gene, PLEC gene, autosomal recessive

## Abstract

Epidermolysis bullosa (EB) is a genetic skin disorder that shows heterogeneous clinical fragility. The patients develop skin blisters congenitally or in the early years of life at the dermo-epithelial junctions, including erosions, hyperkeratosis over the palms and soles. The other associated features are hypotrichosis on the scalp, absent or dystrophic nails, and dental anomalies. Molecular diagnosis through whole-exome sequencing (WES) has become one of the successful tool in clinical setups. In this study, three Pakhtun families from the Khyber Pakhtunkhwa province of Pakistan were ascertained. WES analysis of a proband in each family revealed two novel variants (COL17A1: NM_000494.4: c.4041T>G: p.Y1347^*^ and PLEC: NM_201380.3: c.1283_1285delGCT: p.L426del) and one previously known COL17A1: NM_000494.4:c.3067C>T: p.Q1023^*^) variant in homozygous forms. Sanger sequencing of the identified variants confirmed that the heterozygous genotypes of the obligate carriers. The identified variants have not only increased the mutation spectrum of the COL17A1 and PLEC but also confirms their vital role in the morphogenesis of skin and its associated appendages. WES can be used as a first-line diagnostic tool in genetic testing and counselling families from Khyber Pakhtunkhwa, Pakistan.

## Introduction

Epidermolysis bullosa (EB) is a group of skin fragility disorders that exhibit various blisters caused by mechanical trauma disrupting the dermo-epithelial junction. The phenotypes of EB depend upon the location of defect within the skin and its molecular cause to develop blisters and scarring of skin and mucosa. The overall prevalence of EB in the world is about 19.6 per one million live births (1). According to the latest classification of EB, four clinical types have been defined, i.e., (a) EB simplex (EBS), (b) junctional EB (JEB), (c) dystrophic EB (DEB), and (d) Kindler syndrome (2). Clinical diagnosis is based on immunohistochemistry including skin biopsies and, electron microscopy to understand the histopathology of each EB prototype (3, 4).

Pathogenic variations in seven genes including collagen type XVII alpha 1 chain (*COL17A1*; OMIM 113811), integrin subunit alpha 6 (*ITGA6*; OMIM 147556), integrin subunit beta 4 (*ITGA4*; OMIM 147557), laminin subunit alpha 3 (*LAMA3*; OMIM 600805), laminin subunit beta 3 (*LAMB3*; OMIM 150310), laminin subunit gamma 2 (*LAMC3*; OMIM 150292) and plectin (*PLEC*; OMIM 601282) have been identified so far, causing various JEBs, segregating both in autosomal dominant and recessive forms (2). Some variations of these genes may lead to slightly different phenotypes for example *ITGA4* can cause EB with pyloric atresia (OMIM 226730) and EB of hands and feet (OMIM 131800) and *PLEC* can cause limb girdle muscular dystrophy type 17 (LGMD; OMIM 613723) and EB simplex (EBS; OMIM 226670, 616487, and 131950). Linkage analysis followed by candidate gene Sanger sequencing has remained a successful tool for the causative gene identification in EB families (5); however, Next Generation Sequencing (NGS) technologies using Whole Exome Sequencing (WES) or selected gene panels approaches (3, 6) are more accurate, time-saving and economical as compared to targeted Sanger sequencing of selected candidate genes (7).

In this study, we applied the WES approach for the genetic diagnosis of EB patients in three families from Khyber Pakhtunkhwa province of Pakistan.

## Materials and Methods

### Ethical Approval

The study design was planned according to the Declaration of Helsinki. Approval for this research project and the enrolment of the members for the study were attained from Ethical Review.

Committee (ERC) of Institute of Basic Medical Sciences (IBMS), Khyber Medical University (KMU), Peshawar, and Department of Biotechnology, and Genetic Engineering, Kohat University of Science and Technology (KUST), Kohat, Khyber Pakhtunkhwa, Pakistan. Signed informed consent was also obtained from the legal guardians of the patients.

### Study Subjects

**Figure 1:**
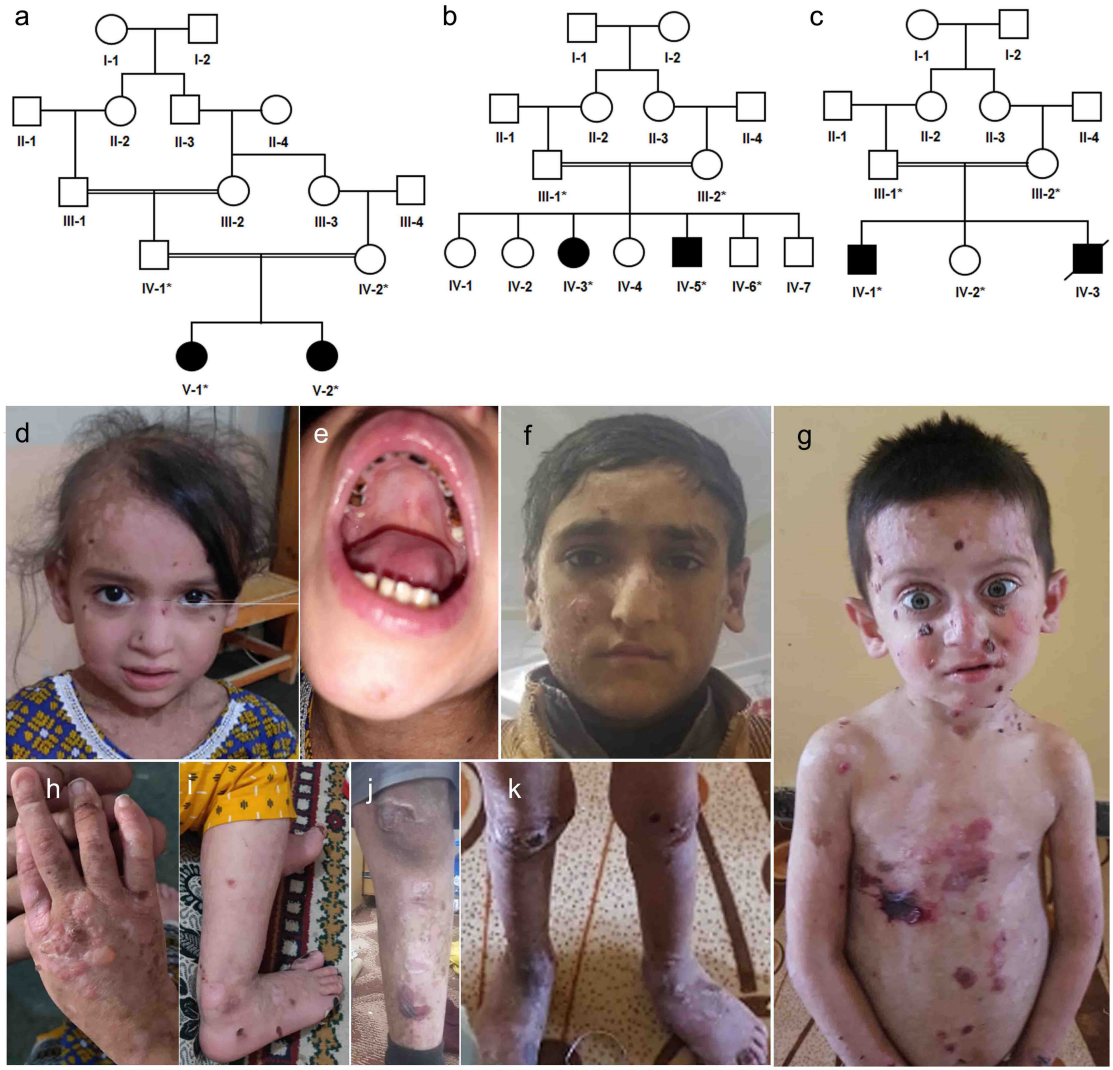
Pedigree analysis and clinical presentation of patients. **(a)** Family A, a five-generation pedigree with two affected siblings in **(b)** family B, a four-generations pedigree with two affected siblings in family B, **(c)** family C, a four-generations pedigree with one affected died and one alive **(d,e,h,i)** clinical presentation of family A affected sibling V-1, **(f,j)** clinical presentation of affected sibling IV-5 in family B, **(g,k)** clinical presentation of affected sibling IV-1 in family C.

Three unrelated consanguineous families from different regions in Khyber Pakhtunkhwa (KP) were recruited. In total, 13 individuals (Figures 1a–c) were available for this study. An expert dermatologist performed detailed clinical examinations of all the affected individuals. Peripheral blood samples were collected in EDTA tubes (BD Vacutainer. K3, Franklin Lakes, NJ, USA). Genomic DNA (gDNA) was extracted from the Whole blood using QIAamp kits (Qiagen, Valencia, CA, USA). The concentration of gDNA was measured on a Nanodrop2000 spectrophotometer. (Thermo Scientific, Schaumburg, IL, USA).

### Whole Exome Sequencing

WES analysis of a proband was performed in each family. Genomic DNA (2 μg) was required for WES target capture and 51 Mb library construction using the SureSelectv7 kit. (Agilent Technologies, Santa Clara., CA, USA). The average sequencing depth was more than 120, and 130 bases long reads were generated. Sequencing reads were run on the Illumina platform (HiSeq 2500, San Diego, CA, USA). Causative variants were filtered and prioritised as described earlier (8). In addition, an affected sibling from each family was analysed for molecular diagnosis in 3billion.io, a commercial company for rare diseases in South Korea.

### Sanger Sequencing

Sanger sequencing was performed using Macrogen Inc. South Korea, to identify the causative variants. Forward and reverse primers (Supplementary Table 1) were used to amplify the target regions. The sequencing data were analysed by aligning against the genomic reference sequence obtained from Ensembl Genome Browser (https://asia.ensembl.org/index.html).

### Three-Dimensional Protein Models of COL17A1 and PLEC1

Homology modelling of COL17A1 was performed through I-Tasser server (9), and the structures were validated through MolProbity server (10). COL17A1 predicted model was subjected to geometry optimization and refinements. Subsequently, the stereochemistry and validity of predicted COL17A1 model was measured through Ramachandran plot that showed 97.8% favoured conformations therefore representing worth of the predicted model stereochemistry. Next, the functional domain characterisation of COL17A1 was predicted using, InterPro (https://www.ebi.ac.uk/interpro), SMART (smart.embl-heidelberg.de/) and Pfam (http://pfam.xfam.org/). The three-dimensional structure of the PLEC plakin domain was retrieved through PDB with PDBID:2ODU and was refined *via* GROMACS available in Chimaera. Molecular dynamics (MD) simulation was performed using standard parameters (11). An appropriate amount of sodium and chloride ions was added to all the systems to neutralise them. The energy minimisation was conducted using the steepest descent method for 5,000 steps and was used for energy minimisation for stable conformations. All MD simulations were performed at 30 ns under constant temperature (300 K) and pressure (1 ATM) with Particle-Mesh Ewald simulation (12) for the analysis of electrostatic interactions. Root mean square deviation (RMSD) and root mean square fluctuation (RMSF) plot of the resulted dynamics trajectories were calculated to assess the stability and fluctuations.

## Results

### Family A

**Figure 2:**
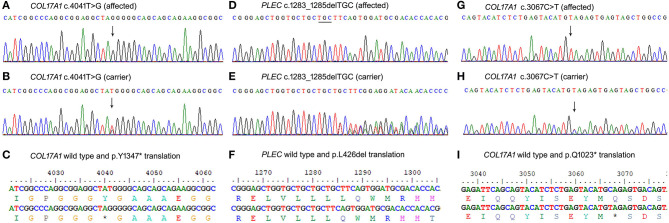
Sanger sequencing results of the three variants identified. **(A–C)** COL17A1 variant c.4041T>G in family A, **(D–F)** PLEC variant c.1283_1285delTGC in family B, and COL17A1 variant c.3067C>T **(G–I)** in family C.

Family A (Figure 1a) had two affected girls (V-1 and V-2) of 6 and 2 years. They were born to cousin marriage with no history of the same disease symptoms in previous ancestral generations. The younger sister (V-2) has mild symptoms of junctional epidermolysis bullosa (JEB), as compared to her elder sister (V-1). The patient (V-1) had generalised growth retardation below the 4th percentile for her age. Skin showed trauma-induced blistering and erythematous bullous associated with crusting and scarring, which diminished with age along with sparse scalp hairs, dystrophic nails, plantar hyperkeratosis, oral mucosal blisters, and hypoplasia of enamel (Figures 1d,e,h,i). She was anaemic, suffering dysphagia, muscle weakness, with a history of recurrent skin, respiratory and urinary tract infections. WES analysis identified a novel non-sense variant in the collagen 17A1 gene (COL17A1: NM_000494.4: c.4041T>G: p.Y1347^*^). Sanger sequencing confirmed autosomal recessive inheritance of the affected allele in the family (Figures 2A–C).

### Family B

Family B (1b) had two affected members (15-year-girl IV-3 and 13-year-boy IV-5), clinically diagnosed of epidermolysis bullosa intermediate form. The patients had fragile blisters and peeling skin, especially on the groyne and leg regions (Figures 1f,j). Their skin showed spontaneous blistering on hands, trunk, and extremities, which healed, leaving scars that become less prominent with time. They also showed hoarseness of voice, dystrophic nails, and plantar hyperkeratosis, along with recurrent skin and respiratory tract infections. WES analysis and subsequent Sanger Sequencing confirmed a novel variant in plectin gene (PLEC: NM_201380.3: c.1283_1285delTGC: p.L426del) (Figures 2D–F). As *PLEC* has been known to cause features other than EB, the members of family were also assessed for muscular dystrophy. Developmental motor milestones were not delayed since birth like sitting, standing, walking and speaking according to age. There was no muscular atrophy and fasciculation on inspection and measuring limb girth and mid-arm circumference. Both active and passive movements plus reflexes were normal. There are no tremors in hands and the gate of the patient was also normal. Serum creatinine phosphokinase (CPK) level measured within the normal ranges.

### Family C

Family C (Figure 1c) had an affected boy of 6 years (IV-1) at the time of this study. Another affected child (IV-3) died in early infancy without any recorded clinical history. The proband IV-1 was clinically diagnosed as an intermediate form of JEB. He had generalised skin blisters affecting the face, hands, trunk, and legs (Figures 1g,k), which decreased in intensity and healed with the development of minimal scars. The patient also exhibited dystrophy of nails, plantar hyperkeratosis, enamel hypoplasia, oral mucosal blisters formation, anaemia, and dysphagia. WES analysis and Sanger sequencing identified a homozygous (COL17A1: NM_000494.4: c.3067C>T: p.Q1023^*^) variant, which creates a premature stop codon in exon 45 (Figures 2G–I).

### Effect of the Three Identified Variations

The variant c.4041T>G creates a premature termination codon at 1,347 amino acids, which is expected to cause loss of normal protein function. A synonymous variant at this position (c.4041T>C; p.Y1347Y) has been reported with a shallow frequency (2.48 ×10 – 5) in the large population cohorts (gnomAD: https://gnomad.broadinstitute.org/). However, the c.4041T>G variant of COL17A1 is novel and has not been reported before. The second homozygous variant c.1283_1285delTGC in PLEC deletes three nucleotides altering the typical reading frame and creates a novel premature termination codon 30 amino acids ahead of the point of deletion. This deletion lies in the non-repeat region and may result in in complete formation of protein leading to aberrant protein function. Similarly, the variant c.3067C>T in COL17A1 creates a stop codon in exon 52. All the three homozygous premature termination codons identified in this study may lead to complete loss of function through the mechanism of non-sense-mediated mRNA decay (13).

### *In-silico* Modelling of COL17A1 and PLEC1 Proteins

**Figure 3:**
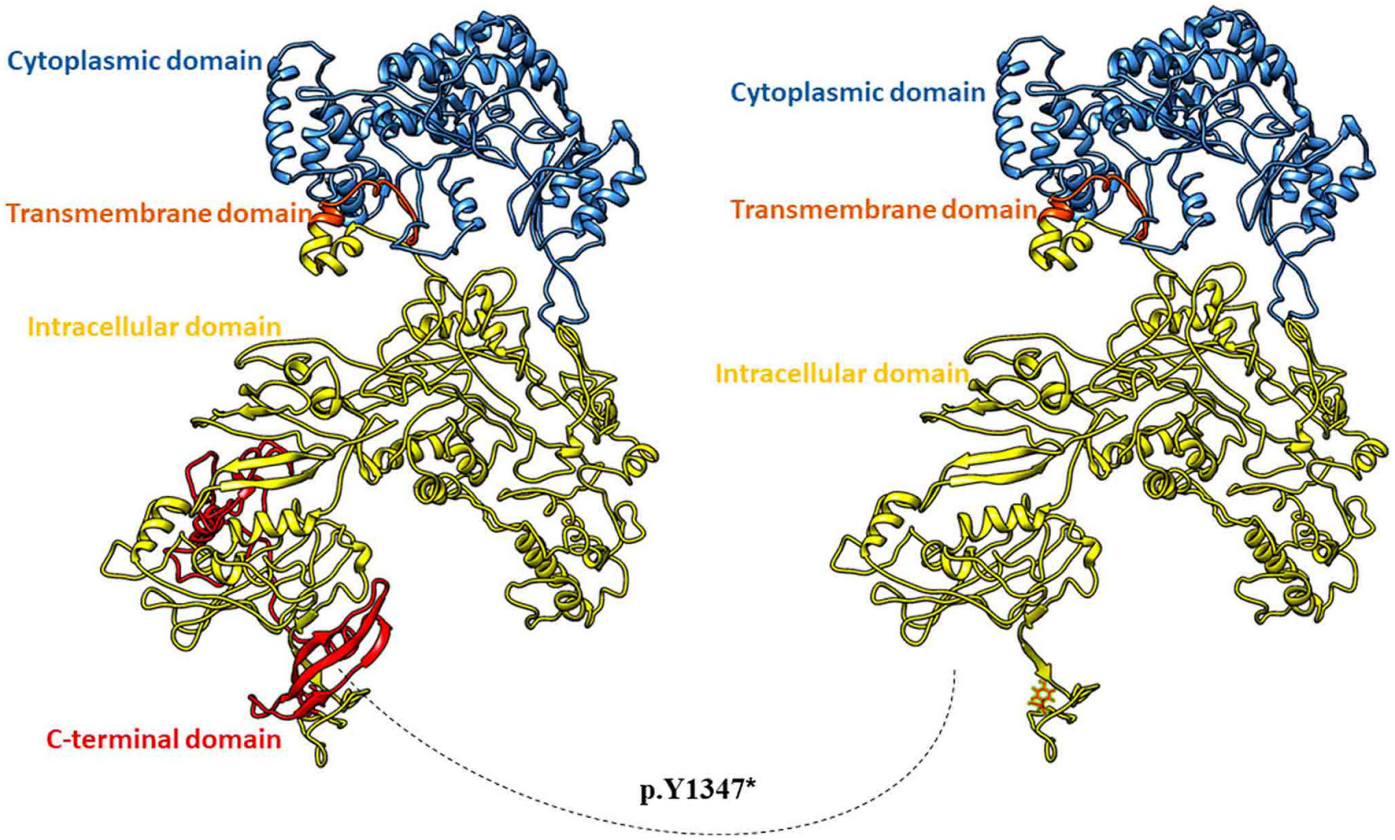
Three-dimensional full-length model of Collagen alpha-1 (XVII). Wild type protein in ribbon model (on the left) and mutant Collagen alpha-1 (XVII) with deletion of c-terminal 151 residues (on the right).

A full-length three-dimensional model of COL17A1 was predicted with a C-score of 0.9 and a Tm score of 0.7. The p.Y1347^*^ was mapped in the ectodomain at the c-terminus of collagen XVII (Figure 3). The premature termination resulted in the deletion of 151 amino acids of the ectodomain and loss of active N-glycosylation site at 1,421 position (Figure 3). N-glycosylation of the ectodermal domain is a vital step in correct plasma membrane trafficking of collagen XVII (14), and deletion of glycosylation site may lead to pathogenic consequences.

**Figure 4:**
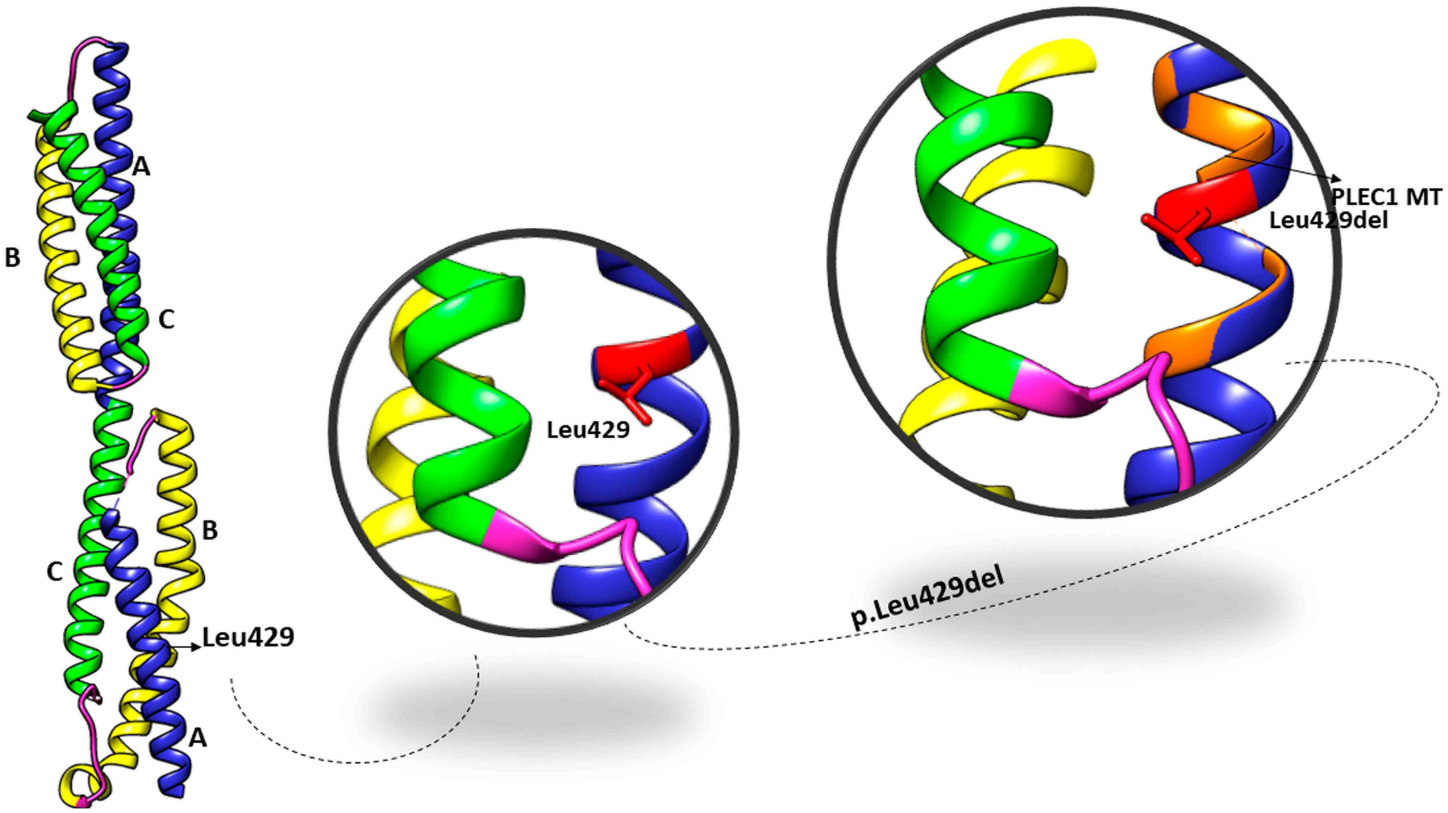
Crystal structure of the plakin domain of PLEC: Domains A, B, and C showed in ribbon representation with equivalent alpha-helices in each domain while Leu429 located in subdomain A shown in zoom views in the superimposition of wild type and mutant PLEC.

**Figure 5:**
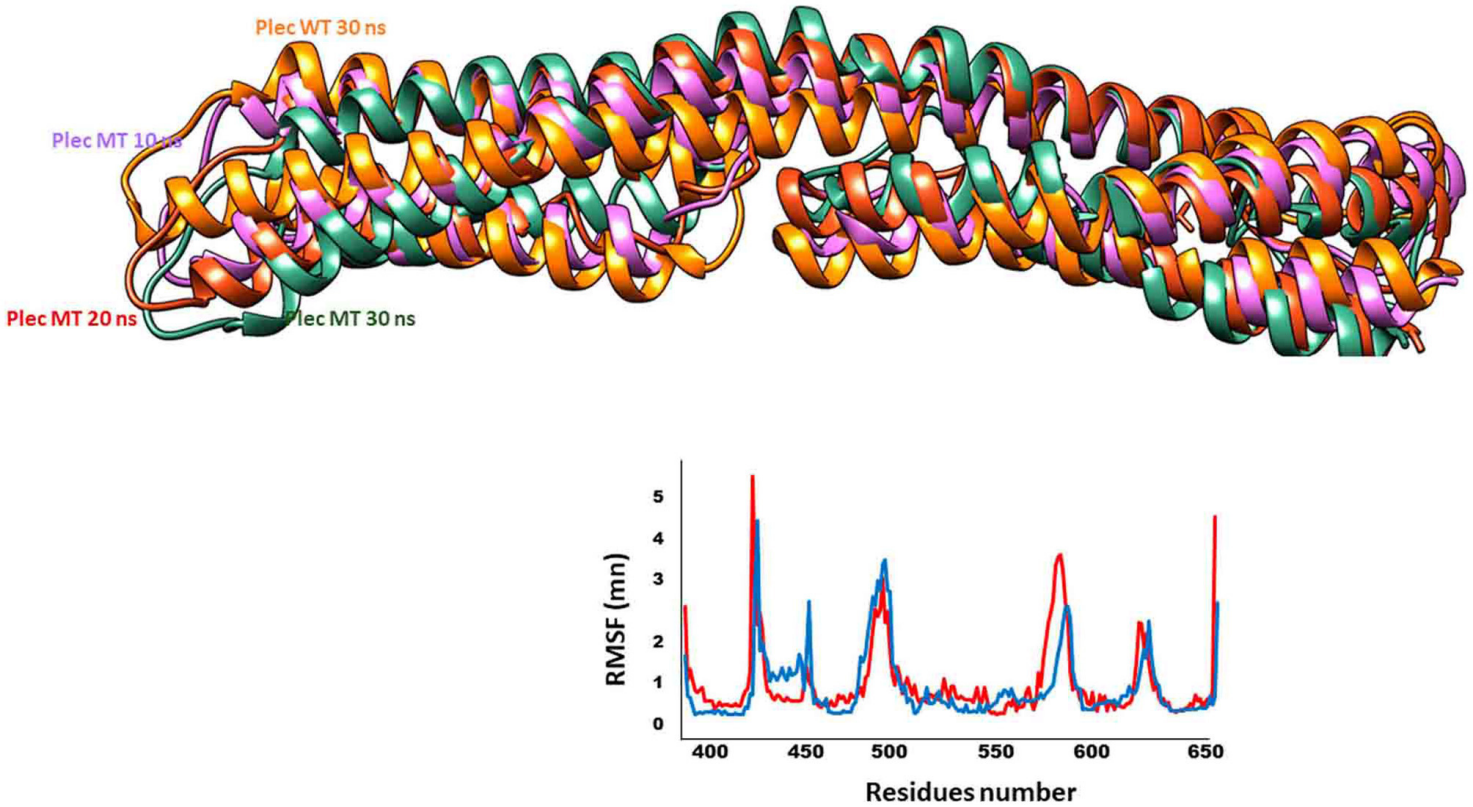
Fluctuations of wild type and mutant PLEC. Dynamic trajectories at different nanoscale shown in respective colours. Note the RMSF plot to show fluctuations of wild type (blue) and mutant (red) PLEC protein.

The PLEC variant (p.L426del) affects the reading frame in the N-terminal globular domain of PLEC1 protein, which harbours actin, and integrin β4 binding domains. The p.Leu426 lies in the plakin domain of PLEC (15), and *via in-silico*, analysis predicted the alteration of p.Leu426del as “deleterious.” The identified non-frameshift deletion variation might affect the binding of PLEC with its binding partners, thus leading to pathological phenotype (Figure 4). MD simulations revealed a high fluctuation among the amino acids of wild type and altered PLEC proteins with reduced hydrophobicity causing disability of the altered protein (Figure 5).

## Discussion

The patients with rare skin malformations, especially those affected with epidermolysis bullosa (EB), have a lifetime of suffering and management complications, especially in the populations of Khyber Pakhtunkhwa, due to lack of genetic testing and non-availability of health facilities. WES has become the first-line genetic test in diagnosing most monogenic diseases worldwide; here in this study, we have used it for a successful molecular diagnosis of EB patients.

The COL17A1 consisting of 56 exons, codes a transmembrane protein, collagen XVII, which is a trimer, of three 180kDa (XVII) chains, with a 466 amino acid intracellular N-terminal domain towards the cytosol, a 23 amino acids transmembrane stretch, and the lamina densa of the basement membrane (15). The detachment of ectodermal domain releases the cells from their cellular binding partners and makes them motile during wound healing and tissue regeneration. COL17A1 is also accountable for follicular stem cell maintenance, cellular migration, and cellular polarity (16).

Clinically the patients in families A and C present moderate to severe blistering tendencies, associated with extra-cutaneous manifestations include dystrophic nails, patchy scarring scalp alopecia, dental anomalies, and compromised life quality, as stated in previous studies (17, 18). The substitutions generate novel non-sense variants (c.4041T>C; p.Y1347Y) and (c.3067C>T: p.Q1023^*^) in the homozygous state, which is expected to cause loss of normal function of protein through non-sense-mediated mRNA decay. The variant c.3067C>T was first reported in heterozygous state (17). Here we report the homozygous genotype of this variant globally and for the first time in the South Asian population.

In Family B, we present a consanguineous Pakistani family presenting epidermolysis bullosa simplex) in two out of seven siblings. Using whole-exome sequencing (WES), we identified a novel homozygous variant (c.1283_1285delTGC; p.Leu426) PLEC. PLEC codes the cytolinker protein plectin, a multi-domain large-sized protein (l > 500 kDa) of the plakin family expressed mainly in the skin, muscle, mucous membranes stratified, and simple squamous epithelia (19). The phenotypic presentation of PLEC genetic variations includes mucocutaneous blistering, muscular dystrophy, pyloric atresia, and cardiomyopathy (19). The plectin is located in the inner plaque of the hemi-desmosomes, at the site of interactions with intermediate filaments (20).

The genetic alterations in PLEC result in several forms of rare human hereditary disorders, collectively known as plectinopathies,” including autosomal recessive EBS with muscular dystrophy (EBS-MD), EBS-MD with myasthenic features (EBS-MD-MyS), limb-girdle muscular dystrophy type2Q (LGMD2Q), EBS with pyloric atresia (EBS-PA), and the autosomal dominant variant EBS-Ogna (19, 21).

In general, the affected individuals exhibit various degrees of clinical phenotypes concerning the severity of blistering of the skin. In addition, in EBS patients, nail deformities, tooth decay, erosive lesions on the oral and laryngeal mucosa, recurrent respiratory and urinary tract infections during infancy have also been reported (22). Features like nails and teeth dystrophies, muscle weakness, recurrent infections are also observed in our patients. In contrast, other features like oral and laryngeal blisters are not detected in the affected individuals reported in this study. *Plec* deficient (-/-) mice model displays blistering of the skin due to keratinocytes degeneration, reduction in the number and stability of hemidesmosomes, skeletal muscle myopathies and the disintegration of intercalated discs' of cardiac muscles and dies 2–3 days after birth due to failure to thrive (23).

This study adds novel variants to the existing pool of *COL17A1* and *PLEC* variants. High frequency of cousin marriages is the main driver of disease risk in the upcoming generations. However, thorough genetic screening helps to reduce the disease burden *via* counselling and premarital planning. Furthermore, the latest technologies of gene editing (CRISPR/Cas9) have the potential to improve the therapeutics in EB patients (24, 25).

## Data Availability Statement

The data presented in the study are deposited in the ClinVar repository, accession number (SCV001739268, SCV001739269).

## Ethics Statement

The studies involving human participants were reviewed and approved by Ethical Review Committee (ERC) of Institute of Basic Medical Sciences (IBMS), Khyber Medical University (KMU), Peshawar, Pakistan. Written informed consent to participate in this study was provided by the participants' legal guardian/next of kin. Written informed consent was obtained from the individual(s), and minor(s)' legal guardian/next of kin, for the publication of any potentially identifiable images or data included in this article.

## Author Contributions

FF, RN, and NW contributed to study design. NB, SAK, and NM contributed to data analysis. NS, SK, and MJ contributed to data generation and analysis. MJ, SK, and NW contributed to writing and finalising the draught. All authors contributed to the article and approved the submitted version.

## Conflict of Interest

The authors declare that the research was conducted in the absence of any commercial or financial relationships that could be construed as a potential conflict of interest.

## Publisher's Note

All claims expressed in this article are solely those of the authors and do not necessarily represent those of their affiliated organizations, or those of the publisher, the editors and the reviewers. Any product that may be evaluated in this article, or claim that may be made by its manufacturer, is not guaranteed or endorsed by the publisher.

## References

[1] Fine JD. Epidemiology of inherited epidermolysis bullosa based on incidence and prevalence estimates from the national epidermolysis bullosa registry. JAMA Dermatol. (2016) 152:1231–8. 10.1001/jamadermatol.2016.2473 27463098

[2] Has C Bauer JW Bodemer C Bolling MC Bruckner-Tuderman L Diem A. Consensus reclassification of inherited epidermolysis bullosa and other disorders with skin fragility. Br J Dermatol. (2020) 183:614–27. 10.1111/bjd.18921 32017015

[3] Rossi S Castiglia D Pisaneschi E Diociaiuti A Stracuzzi A Cesario C. Immunofluorescence mapping, electron microscopy and genetics in the diagnosis and sub-classification of inherited epidermolysis bullosa: a single-center retrospective comparative study of 87 cases with long-term follow-up. J Eur Acad Dermatol Venereol. (2021) 35:1007–16. 10.1111/jdv.17060 33274474

[4] Yenamandra VK Bhari N Ray SB Sreenivas V Dinda AK Scaria V. Diagnosis of inherited epidermolysis bullosa in resource-limited settings: immunohistochemistry revisited. Dermatology. (2017) 233:326–32. 10.1159/000478856 29069641

[5] Pulkkinen L Uitto J. Mutation analysis and molecular genetics of epidermolysis bullosa. Matrix Biol. (1999) 18:29–42. 10.1016/S0945-053X(98)00005-5 10367729

[6] Takeichi T Liu L Fong K Ozoemena L McMillan JR Salam A. Whole-exome sequencing improves mutation detection in a diagnostic epidermolysis bullosa laboratory. Br J Dermatol. (2015) 172:94–100. 10.1111/bjd.13190 24947307

[7] Bardhan A Bruckner-Tuderman L Chapple ILC Fine JD Harper N Has C. Epidermolysis bullosa. Nat Rev Dis Primers. (2020) 6:78. 10.1038/s41572-020-0210-0 32973163

[8] Serafi R Jelani M Almramhi MM Mohamoud HSA Ahmed S Alkhiary YM. Identification of two homozygous sequence variants in the COL7A1 gene underlying dystrophic epidermolysis bullosa by whole-exome analysis in a consanguineous family. Ann Hum Genet. (2015) 79:350–6. 10.1111/ahg.12123 26102279

[9] Zhang Y. I-TASSER server for protein 3D structure prediction. BMC *Bioinformatics*. (2008) 9:40. 10.1186/1471-2105-9-40 18215316 PMC2245901

[10] Chen VB Arendall WB III Headd JJ Keedy DA Immormino RM Kapral GJ. MolProbity: all-atom structure validation for macromolecular crystallography. Acta crystallographica Section D. Biol Crystallogr. (2010) 66:12–21. 10.1107/S0907444909042073 PMC2803126 20057044

[11] Abraham MJ Murtola T Schulz R Páll S Smith JC Hess B. GROMACS: high performance molecular simulations through multi-level parallelism from laptops to supercomputers. SoftwareX. (2015) 1-2:19–25. 10.1016/j.softx.2015.06.001

[12] Essman U Perera L Berkowtity ML Darden T Lee H. A smooth particle mesh Ewald method. J Chem Phys. (1995) 103:8577. 10.1063/1.47011727760290

[13] Peltz SW Brown AH Jacobson A. mRNA destabilization triggered by premature translational termination depends on at least three cis-acting sequence elements and one trans-acting factor. Genes Dev. (1993) 7:1737–54. 10.1101/gad.7.9.1737 8370523

[14] Franzke CW Has C Schulte C Huilaja L Tasanen K Aumailley M. C-terminal truncation impairs glycosylation of transmembrane collagen XVII and leads to intracellular accumulation. J Bio Chem. (2006) 281:30260–8. 10.1074/jbc.M604464200 16899459

[15] Pagel KA Antaki D Lian A Mort M Cooper DN Sebat J. Pathogenicity and functional impact of non-frameshifting insertion/deletion variation in the human genome. PLoS Comp Bio. (2019) 15:e1007112. 10.1371/journal.pcbi.1007112 31199787 PMC6594643

[16] Watanabe M Natsuga K Nishie W Kobayashi Y Donati G Suzuki S. Type XVII collagen coordinates proliferation in the interfollicular epidermis. Elife. (2017) 6:e26635. 10.7554/eLife.26635 28693719 PMC5505703

[17] Gatalica B Pulkkinen L Li K Kuokkanen K Ryynänen M McGrath JA. Cloning of the human type XVII collagen gene (COL17A1), and detection of novel mutations in generalized atrophic benign epidermolysis bullosa. Am J Hum Genet. (1997) 60:352–65. 9012408 PMC1712405

[18] Nakano A Lestringant GG Paperna T Bergman R Gershoni R Frossard P. Junctional epidermolysis bullosa in the Middle East: clinical and genetic studies in a series of consanguineous families. J Am Acad Dermatol. (2002) 46:510–6. 10.1067/mjd.2002.119673 11907499

[19] Winter L Wiche G. The many faces of plectin and plectinopathies: pathology and mechanisms. Acta Neuropathol. (2013) 125:77–93. 10.1007/s00401-012-1026-0 22864774

[20] Smith FJ Eady RA Leigh IM McMillan JR Rugg EL Kelsell DP. Plectin deficiency results in muscular dystrophy with epidermolysis bullosa. Nat Genet. (1996) 13:450–7. 10.1038/ng0896-450 8696340

[21] Elliott CE Becker B Oehler S Castañón MJ Hauptmann R Wiche G. Plectin transcript diversity: identification and tissue distribution of variants with distinct first coding exons and rodless isoforms. Genomics. (1997) 42:115–25. 10.1006/geno.1997.4724 9177781

[22] Babić I Karaman-Ilić M Pustisek N Susić S Skarić I Kljenak A. Respiratory tract involvement in a child with epidermolysis bullosa simplex with plectin deficiency: a case report. Int J Pediatr Otorhinolaryngol. (2010) 74:302–5. 10.1016/j.ijporl.2009.10.002 20044146

[23] Andrä K Lassmann H Bittner R Shorny S Fässler R Propst F. Targeted inactivation of plectin reveals essential function in maintaining the integrity of skin, muscle, and heart cytoarchitecture. Genes Dev. (1997) 11:3143–56. 10.1101/gad.11.23.3143 9389647 PMC316746

[24] Hainzl S Peking P Kocher T Murauer EM Larcher F Del Rio M. COL7A1 editing *via* CRISPR/Cas9 in recessive dystrophic epidermolysis bullosa. Mol Ther. (2017) 25:2573–84. 10.1016/j.ymthe.2017.07.005 28800953 PMC5675435

[25] Izmiryan A Danos O Hovnanian A. Meganuclease-mediated COL7A1 gene correction for recessive dystrophic epidermolysis bullosa. J Invest Dermatol. (2016) 136:872–5. 10.1016/j.jid.2015.11.028 26897595

