# Supplementary material for: Whole Exome Sequencing Confirms Molecular Diagnostics of Three Pakhtun Families With Autosomal Recessive Epidermolysis Bullosa
Source: Front Pediatr. 2021 Aug 3;9:727288. doi: 10.3389/fped.2021.727288 (PMC8369263; doi:10.3389/fped.2021.727288)
Supplement: Supplementary file 1 [file Table_1.docx]

**Supplementry Table S1:** List of *COL17A1* premature termination codons identified so far

| **No** | **Change** | | **Exon** | **Phenotype** | **Reference** |
| --- | --- | --- | --- | --- | --- |
|  | c.DNA | Protein |  |  |  |
| 1 | c.25C>T | p.R9* | 2 | JEB | (Pfendner et al. 2003) |
| 2 | c.433C>T | p.R145* | 8 | JEB | (Tasanen et al. 2000) |
| 3 | c.460C>T | p.R154* | 8 | JEB-gen | (Kiritsi et al. 2011) |
| 4 | c.505C>T | p.R169* | 9 | JEB-loc | (Kiritsi et al. 2011) |
| 5 | c.772G>T | p.G258* | 11 | EB-atrophic benign | (Shimizu et al. 1998) |
| 6 | c.997C>T | p.Q333* | 14 | JEB | (Vahidnezhad et al. 2017) |
| 7 | c.1392G>A | p.W464* | 17 | JEB | (Kiritsi et al. 2011) |
| 8 | c.1395G>A | p.W465* | 17 | EB-Herlitz | (Varki et al. 2006) |
| 9 | c.1646G>A | p.W549* | 18 | AI-nonsyndromic | (Prasad et al. 2016) |
| 10 | c.1696C>T | p.R566* | 19 | EB-Herlitz | (Varki et al. 2006) |
| 11 | c.1873C>T | p.R625* | 23 | AI-nonsyndromic | (Prasad et al. 2016) |
| 12 | c.2062C>T | p.R688* | 26 | JEB | (Vahidnezhad et al. 2019) |
| 13 | c.2251C>T | p.Q751* | 30 | JEB | (Pasmooij et al. 2004) |
| 14 | c.2383C>T | p.R795* | 33 | JEB-non Herlitz | (Ruzzi et al. 2001) |
| 15 | c.2407G>T | p.G803* | 34 | EB-atrophic benign | (Darling et al. 1997) |
| 16 | c.2564T>G | p.L855* | 37 | JEB | (Floeth and Bruckner-Tuderman 1999) |
| 17 | c.3046C>T | p.Q1016* | 45 | JEB | (Schumann et al. 1997) |
| **18** | **c.3067C>T** | **p.Q1023*** | **45** | **EB** | **(Gatalica et al. 1997) This study** |
| 19 | c.3579G>A | p.W1193* | 50 | JEB | (Vahidnezhad et al. 2017) |
| 20 | c.3676C>T | p.R1226* | 51 | EB | (McGrath et al. 1995) |
| **21** | **c.4041T>G** | **p.Y1347*** | **52** | **JEB** | **This study** |
| 22 | c.4159C>T | p.Q1387* | 53 | JEB | (Nakamura et al. 2006) |
| 23 | c.4207C>T | p.Q1403* | 53 | EB-atrophic benign | (Darling et al. 1997) |

^EB, epidermolysis bollusa; JEB, junctional epidermolysis bollusa; JEB-gen, generalized junctional epidermolysis bollusa; JEB-loc, localized junctional epidermolysis bollusa; AI, amylogenesis imperfecta^

**References**

Darling TN, McGrath JA, Yee C, Gatalica B, Hametner R, Bauer JW, Pohla-Gubo G, Christiano AM, Uitto J, Hintner H, Yancey KB (1997) Premature termination codons are present on both alleles of the bullous pemphigoid antigen 2/type XVII collagen gene in five Austrian families with generalized atrophic benign epidermolysis bullosa. J Invest Dermatol 108: 463-8. doi: 10.1111/1523-1747.ep12289718

Floeth M, Bruckner-Tuderman L (1999) Digenic junctional epidermolysis bullosa: mutations in COL17A1 and LAMB3 genes. Am J Hum Genet 65: 1530-7. doi: 10.1086/302672

Gatalica B, Pulkkinen L, Li K, Kuokkanen K, Ryynänen M, McGrath JA, Uitto J (1997) Cloning of the human type XVII collagen gene (COL17A1), and detection of novel mutations in generalized atrophic benign epidermolysis bullosa. Am J Hum Genet 60: 352-65.

Kiritsi D, Kern JS, Schumann H, Kohlhase J, Has C, Bruckner-Tuderman L (2011) Molecular mechanisms of phenotypic variability in junctional epidermolysis bullosa. J Med Genet 48: 450-7. doi: 10.1136/jmg.2010.086751

McGrath JA, Gatalica B, Christiano AM, Li K, Owaribe K, McMillan JR, Eady RA, Uitto J (1995) Mutations in the 180-kD bullous pemphigoid antigen (BPAG2), a hemidesmosomal transmembrane collagen (COL17A1), in generalized atrophic benign epidermolysis bullosa. Nat Genet 11: 83-6. doi: 10.1038/ng0995-83

Nakamura H, Sawamura D, Goto M, Nakamura H, Kida M, Ariga T, Sakiyama Y, Tomizawa K, Mitsui H, Tamaki K, Shimizu H (2006) Analysis of the COL17A1 in non-Herlitz junctional epidermolysis bullosa and amelogenesis imperfecta. Int J Mol Med 18: 333-7.

Pasmooij AM, van Zalen S, Nijenhuis AM, Kloosterhuis AJ, Zuiderveen J, Jonkman MF, Pas HH (2004) A very mild form of non-Herlitz junctional epidermolysis bullosa: BP180 rescue by outsplicing of mutated exon 30 coding for the COL15 domain. Exp Dermatol 13: 125-8. doi: 10.1111/j.0906-6705.2004.00141.x

Pfendner EG, Nakano A, Pulkkinen L, Christiano AM, Uitto J (2003) Prenatal diagnosis for epidermolysis bullosa: a study of 144 consecutive pregnancies at risk. Prenat Diagn 23: 447-56. doi: 10.1002/pd.619

Prasad MK, Geoffroy V, Vicaire S, Jost B, Dumas M, Le Gras S, Switala M, Gasse B, Laugel-Haushalter V, Paschaki M, Leheup B, Droz D, Dalstein A, Loing A, Grollemund B, Muller-Bolla M, Lopez-Cazaux S, Minoux M, Jung S, Obry F, Vogt V, Davideau JL, Davit-Beal T, Kaiser AS, Moog U, Richard B, Morrier JJ, Duprez JP, Odent S, Bailleul-Forestier I, Rousset MM, Merametdijan L, Toutain A, Joseph C, Giuliano F, Dahlet JC, Courval A, El Alloussi M, Laouina S, Soskin S, Guffon N, Dieux A, Doray B, Feierabend S, Ginglinger E, Fournier B, de la Dure Molla M, Alembik Y, Tardieu C, Clauss F, Berdal A, Stoetzel C, Manière MC, Dollfus H, Bloch-Zupan A (2016) A targeted next-generation sequencing assay for the molecular diagnosis of genetic disorders with orodental involvement. J Med Genet 53: 98-110. doi: 10.1136/jmedgenet-2015-103302

Ruzzi L, Pas H, Posteraro P, Mazzanti C, Didona B, Owaribe K, Meneguzzi G, Zambruno G, Castiglia D, D'Alessio M (2001) A homozygous nonsense mutation in type XVII collagen gene (COL17A1) uncovers an alternatively spliced mRNA accounting for an unusually mild form of non-Herlitz junctional epidermolysis bullosa. J Invest Dermatol 116: 182-7. doi: 10.1046/j.1523-1747.2001.00229.x

Schumann H, Hammami-Hauasli N, Pulkkinen L, Mauviel A, Küster W, Lüthi U, Owaribe K, Uitto J, Bruckner-Tuderman L (1997) Three novel homozygous point mutations and a new polymorphism in the COL17A1 gene: relation to biological and clinical phenotypes of junctional epidermolysis bullosa. Am J Hum Genet 60: 1344-53. doi: 10.1086/515463

Shimizu H, Takizawa Y, Pulkkinen L, Zone JJ, Matsumoto K, Saida T, Uitto J, Nishikawa T (1998) The 97 kDa linear IgA bullous dermatosis antigen is not expressed in a patient with generalized atrophic benign epidermolysis bullosa with a novel homozygous G258X mutation in COL17A1. J Invest Dermatol 111: 887-92. doi: 10.1046/j.1523-1747.1998.00363.x

Tasanen K, Floeth M, Schumann H, Bruckner-Tuderman L (2000) Hemizygosity for a glycine substitution in collagen XVII: unfolding and degradation of the ectodomain. J Invest Dermatol 115: 207-12. doi: 10.1046/j.1523-1747.2000.00049.x

Vahidnezhad H, Youssefian L, Saeidian AH, Touati A, Sotoudeh S, Abiri M, Barzegar M, Aghazadeh N, Mahmoudi H, Norouz-Zadeh S, Hamid M, Zahabiyon M, Bagherian H, Zeinali S, Fortina P, Uitto J (2017) Multigene Next-Generation Sequencing Panel Identifies Pathogenic Variants in Patients with Unknown Subtype of Epidermolysis Bullosa: Subclassification with Prognostic Implications. J Invest Dermatol 137: 2649-2652. doi: 10.1016/j.jid.2017.07.830

Vahidnezhad H, Youssefian L, Saeidian AH, Zeinali S, Touati A, Abiri M, Sotoudeh S, Norouz-Zadeh S, Amirinezhad N, Mozafari N, Daneshpazhooh M, Mahmoudi H, Hamid M, Bradfield JP, Kim CE, Hakonarson H, Uitto J (2019) Genome-wide single nucleotide polymorphism-based autozygosity mapping facilitates identification of mutations in consanguineous families with epidermolysis bullosa. Exp Dermatol 28: 1118-1121. doi: 10.1111/exd.13501

Varki R, Sadowski S, Pfendner E, Uitto J (2006) Epidermolysis bullosa. I. Molecular genetics of the junctional and hemidesmosomal variants. J Med Genet 43: 641-52. doi: 10.1136/jmg.2005.039685
