# Supplementary figures and images for: Whole Exome Sequencing Confirms Molecular Diagnostics of Three Pakhtun Families With Autosomal Recessive Epidermolysis Bullosa
Source: Front Pediatr. 2021 Aug 3;9:727288. doi: 10.3389/fped.2021.727288 (PMC8369263; doi:10.3389/fped.2021.727288)

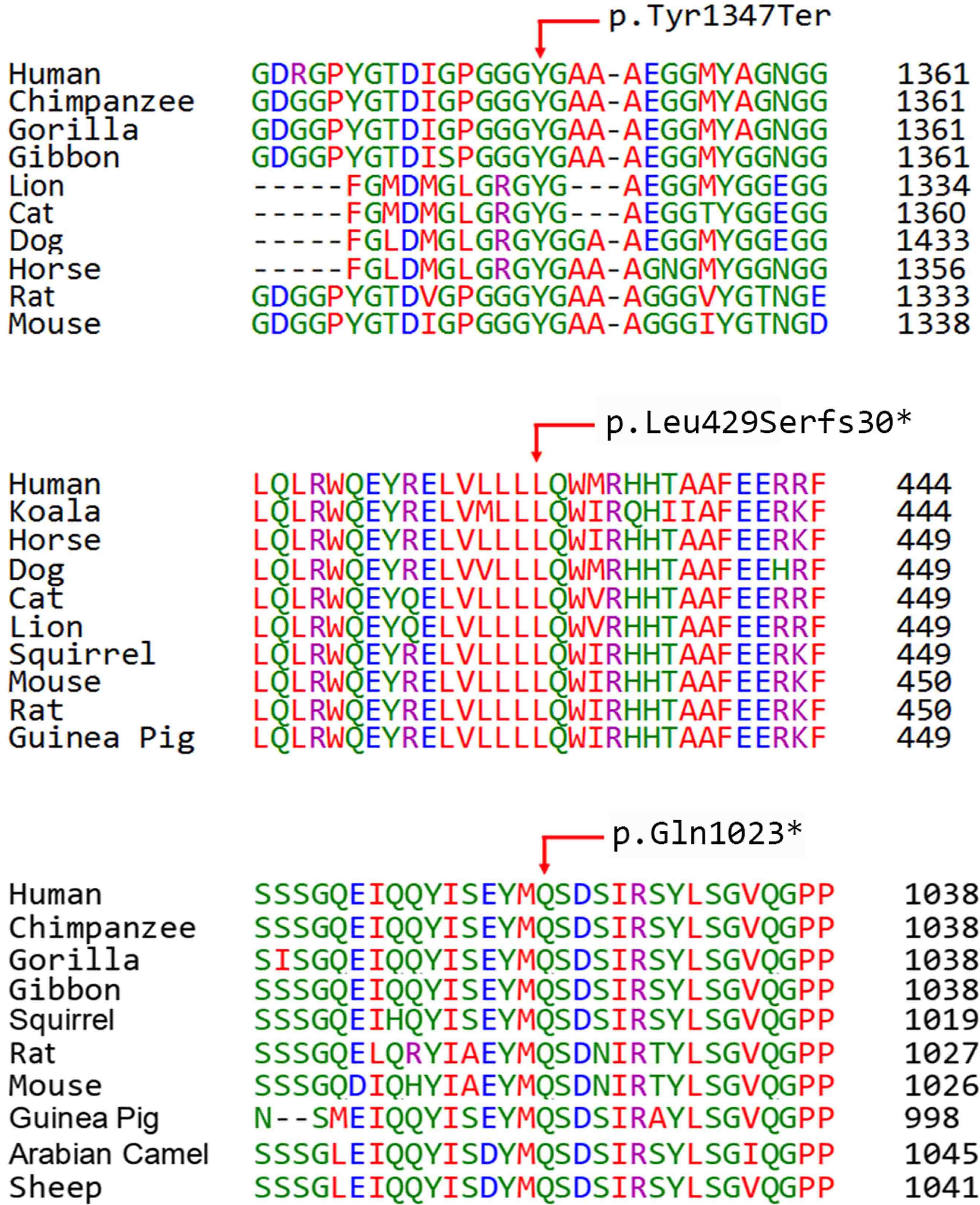

Supplement: Supplementary file 2 [file Image_1.JPEG]
